# How advances in low-g plumbing enable space exploration

**DOI:** 10.1038/s41526-022-00201-y

**Published:** 2022-05-20

**Authors:** M. M. Weislogel, J. C. Graf, A. P. Wollman, C. C. Turner, K. J. T. Cardin, L. J. Torres, J. E. Goodman, J. C. Buchli

**Affiliations:** 1 Portland State University, 1930 SW 4th Ave, Portland, OR 97102 USA; 2 IRPI LLC, 27501 SW 95th Ave, Suite 930, Wilsonville, OR 97070-5705 USA; 3 NASA Johnson Space Center, 2101 E. NASA Pkwy, Houston, TX 77058 USA

**Keywords:** Fluid dynamics, Aerospace engineering

## Abstract

In many ways, plumbing is essential to life support. In fact, the advance of humankind on Earth is directly linked to the advance of clean, healthy, reliable plumbing solutions. Shouldn’t this also be true for the advancement of humankind in space? Unfortunately, the reliability of even the simplest plumbing element aboard spacecraft is rarely that of its terrestrial counterpart. This state of affairs is due entirely to the near-weightless “low-g” state of orbiting and coast spacecraft. But the combined passive capillary effects of surface tension, wetting, and system geometry in space can be exploited to replace the passive role of gravity on earth, and thus achieve similar outcomes there. In this paper, we review a selection of experiments conducted in low-g environments (i.e., ISS and drop towers) that focus on capillary fluidic phenomena. The results of each experiment are highly applicable to subsequent advances in spacecraft plumbing. With examples ranging from spurious droplet ejections to passive bubble coalescence, to droplet bouncing, to complex container wicking, we show how simple low-g demonstrations can lead to significant reliability improvements in practical passive plumbing processes from pipetting to liquid-gas separations, to wastewater transport, to drinking in space.

## Introduction

Most terrestrial plumbing systems would fail entirely without the ever-present passive assistance of gravity. Appropriate examples from the bathroom include the sink, the shower, and the toilet. Appropriate examples from the kitchen include the dishwasher, the disposal, and the coffee maker. Gravity-driven drainage is what makes these systems work—bubbles rise, droplets fall. Clearly, such terrestrial plumbing systems would work flawlessly aboard orbiting and coast spacecraft if we simply matched the gravitational acceleration environment with a centrifugal (or linear) acceleration environment creating artificial gravity. We often do this aboard spacecraft to separate liquids from gases^1^, separate solids from liquids^2^, and weigh humans^3^. However, compared to the simplicity of the terrestrial plumbing system, the equivalent rotating spacecraft system tends to be somewhat cumbersome, with noisy moving parts, challenging seals, an electric power draw penalty, and increased cost. Despite historical successes in spaceflight, the complexity of such solutions produces plumbing systems in space that are significantly less reliable than those on earth. A case in point is the current ISS toilet. The approximately $19M device costs millions per year to maintain and requires at least six maintenance operations/repairs per year^4^. A new $23M 10-year life toilet is under development^5^. Contrast these performance metrics to a typical terrestrial toilet with a one-time installed cost of less than $500, service of as little as once every 10 years, and life expectancy up to 50 years^6^. Regarding toilets, from an order of magnitude perspective, thanks to gravity, terrestrial plumbing outperforms spacecraft plumbing by a factor of ~5 for life expectancy, a factor of ~60 for reliability and maintenance, and a factor of ~40,000 for costs.

It may be some time before we can reproduce the reliability of terrestrial plumbing aboard spacecraft. However, during the present ISS era, numerous fluid physics capillary flow-focused flight experiments and technology demonstrations have been conducted, the accumulative results of which are making it increasingly possible to meet advanced spacecraft fluid system (“plumbing”) requirements with increasingly passive predominately capillary solutions—all in hopes of achieving more earth-like reliability. The spacecraft fluid systems most likely to benefit from the recent progress are pervasive and include both engineering and life support functions such as (1) storable and cryogenic liquid propellants and fuels management, (2) thermal fluids management for environment control, (3) the collection, purification, and recycling of aqueous streams for the crew, plant, and animal habitats, and (4) countless fluids handling operations required of physical science experiments, food preparation, laundry, waste disposal, medical procedures, wet lab unit operations, and more. The fundamental low-g capillary fluidics challenges associated with these systems include efficient prediction of (1) liquid interface position and meta-stability, (2) capillary flows in complex geometries, (3) multiphase flows, transport, and phase change heat transfer, and (4) the theoretical and numerical boundary conditions and models to predict them. Such applied and fundamental challenges for real fluids have long been identified by NASA and the aerospace community^7,8^.

## Scope

In this paper, we use the term “capillary solutions” or “capillary fluidics solutions” to describe often low-tech plumbing solutions that exploit the combined passive effects of surface tension, wetting, and system geometry as a replacement to the passive acceleration of gravity. Single-phase flow phenomena are largely the same on earth as in space. The challenges arise in space when two or more phases of differing densities are present (i.e., gas-liquid flows). Drawing primarily from projects we have worked on directly, we highlight several uncomplicated yet persistently problematic spacecraft fluids applications in need of reliable capillary fluidic solutions. We are seeking solutions with a level of simplicity and reliability on par with equivalent terrestrial solutions; namely, passive systems without moving parts. The perhaps strange examples we select share the common story of how scientific research on earth and in space is leading to technological advancements in plumbing aboard spacecraft—plumbing solutions that are actually, though perhaps subtly, enabling the human exploration of space. It often requires decades for fundamental research to produce practical benefits^9^. October 31, 2020 marked the completion of the International Space Station’s second decade in space. As regards plumbing in space, it appears we are on pace with the marked increase in capillary fluidic solutions that is currently underway.

## Four example capillary fluidics solutions for advanced spacecraft plumbing

### Coffee cups, fuel tanks, and well plates

In the absence of gravity, there is no up or down. Nothing floats. There is no top or bottom. Where the coffee is in your cup is a good question to ask, so is how you plan to drink it once you get it in the cup. Changing the drink can change the outcome. As mentioned above, the combined effects of surface tension, wetting conditions, and container shape can be exploited to passively solve this capillary fluidic plumbing problem when gravity is negligible.

**Fig. 1:**
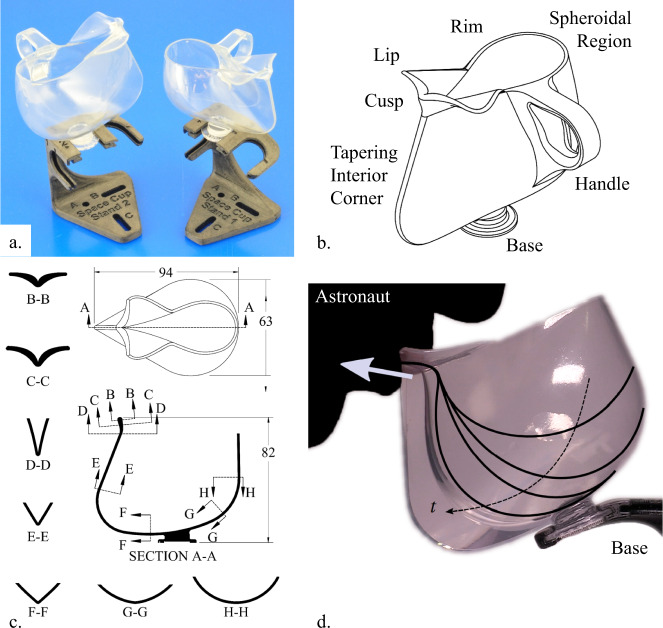
The Capillary Beverage experiment. **a** Image of the space cup (left), space cup demitasse (right), and stands, **b** solid model with labels, **c** sequence of tapering sections, and **d** water surface profiles (black lines) during continuous drinking by astronaut S. Kelly on ISS in time *t* with a large arrow indicating average liquid flow rate and direction.

**Fig. 2:**
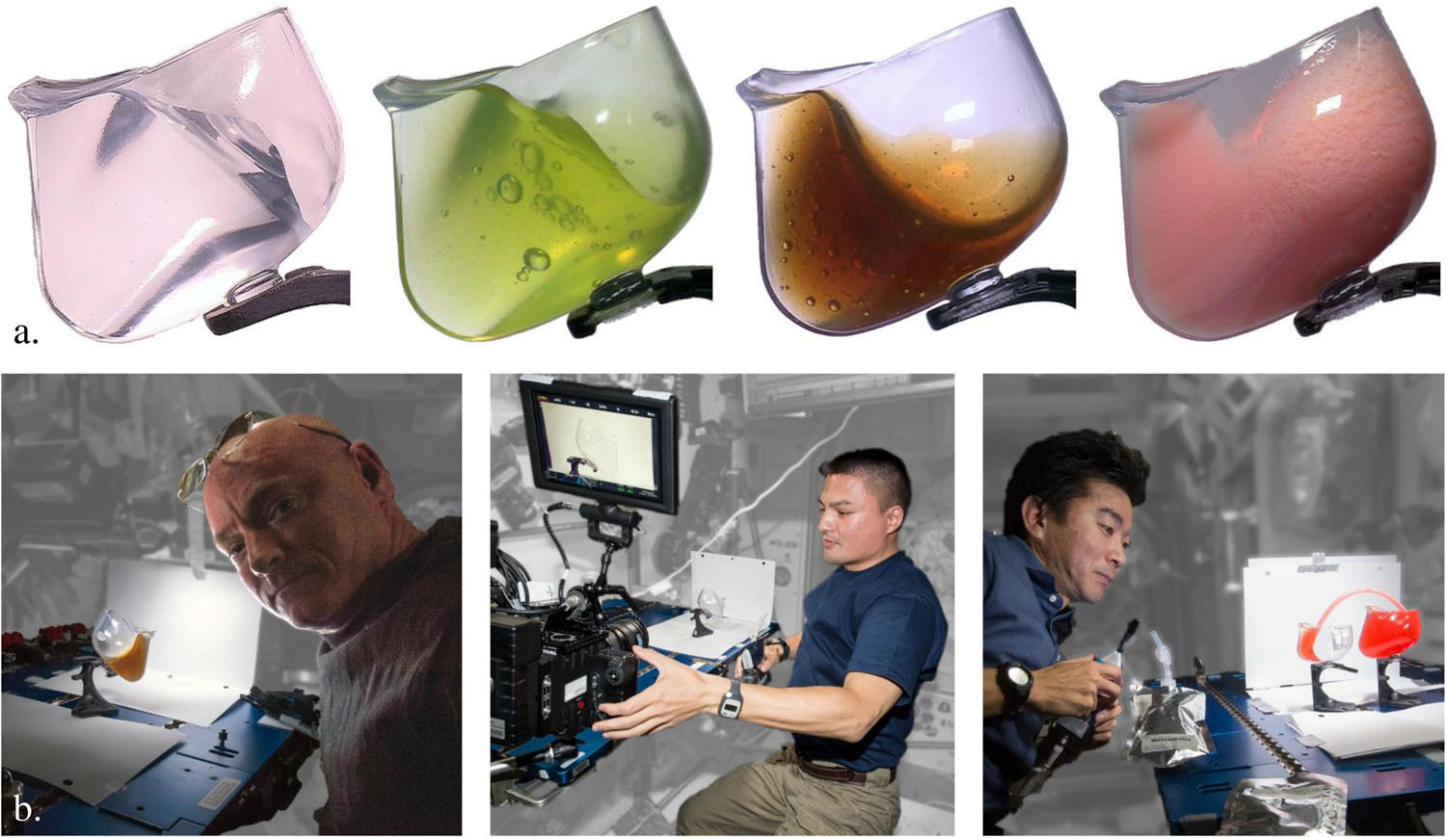
The Capillary Beverage experiment “science”. **a** Space cup with various beverages: bubble-free water, limeade, Kona Black coffee with small bubbles and undissolved coffee particles, and peach-mango smoothie. **b** NASA astronauts S. Kelly, K. Lindgren, and JAXA astronaut K. Yui on ISS.

After initial demonstrations of in situ fabricated prototypes by astronaut Don Pettit^10^ in August 2014, an “engineered” capillary fluidic cup was proposed to NASA for flight demonstration aboard ISS. Under the operational name “Capillary Beverage” experiment^11^, six 3D printed cups were flight certified and launched to ISS, with onboard demonstrations conducted during the May–August 2015 time frame. Images of two of the “Space Cups” are provided in Fig. 1, which includes a solid model identifying key capillary fluidic features. So that the astronauts can drink aromatic beverages from the cup as if on earth, the geometric design produces a continuous capillary pressure gradient toward the lip of the cup. To do so, the spheroidal portion of the cup lofts into an interior corner that tapers into a cusp at the lip as shown in Fig. 1c. The stability of the liquid surface to manually imparted perturbations during handling is enhanced by the enveloping rim. As shown in Fig. 2, by design, the lip of the cup is the “bottom” of the cup, and when the astronaut makes a connection at the lip, the liquid is spontaneously wicked into the mouth, the crew regulating the rate by mouth shape and suction. The entire contents of the cup may be drained this way regardless of the wetting condition of the liquid as exemplified in Fig. 1d for water during continuous drinking in space. Small fast spurious-free droplets are created when the cup is detached at the lip as will be described in Section Rivulet rupture, pipetting, and slime in space. Large bubbles in the liquid are passively exuded from the liquid during the flow process as will be described in Section Low-g flows in sealed chambers, open channels, and in-line bubble phase separators. Further details of the Capillary Beverage experiments’ static and dynamic quantitative verifications, numerical predictions, and comparisons are reported by Wollman^12^.

**Fig. 3:**
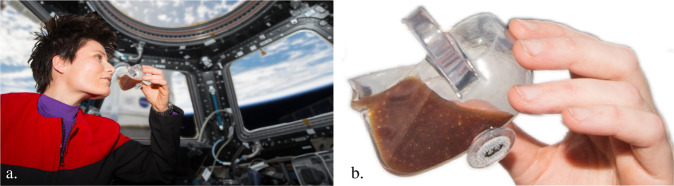
Espresso in the demitasse Space Cup. **a** Italian ESA astronaut S. Cristoforetti drinking ISSpresso^14^ espresso from demitasse Space Cup in the cupola of the ISS. **b** Magnified view of espresso located at cup lip with crema distributed throughout (courtesy NASA).

At least coffee, cocoa, fruit punch, limeade, water, and peach-mango smoothie were successfully imbibed using the cups during the entertaining demonstrations of US astronauts Scott Kelly, Kjell Lindgren, and JAXA astronaut Kimiya Yui^13^ shown in Fig. 2. ISSpresso^14^ espresso was the first fluid consumed by Italian ESA astronaut Samantha Christoforetti using the demitasse Space Cup, Fig. 1a (right). As shown in Fig. 3, during an extemporaneous activity, Christoforetti dispensed espresso into the cup, flew into the ISS cupola, and consumed it without incident^15^.

For safety and design engineers on the ground, this event was met with initial alarm giving way to muted appreciation. A scalding drink (espresso) was consumed from an open container (Space Cup) in a “clean” spacecraft environment (ISS cupola)—a triple violation. But the nature of the “plumbing” solution of the cup was earth-like enough as to pass with limited concern. The liquid is stably contained within the cup, it is fairly insensitive to perturbations caused by normal handling, it is similarly imbibed as if on earth, it is readily filled and drained for a wide range of liquids, and the crew comments that the process of a “home harkening activity” allows them to actually smell the beverage while drinking it—ISS crews normally drink from sealed bags through straws. From this perspective, the Space Cup provides a simple robust no-moving-parts capillary fluidics solution to a longstanding low-g plumbing problem. In other tests on ISS, it was shown that forced spills of the cup in orbit are easier to contain than similar spills on earth. It is thus safer to drink coffee next to your computer in space than on the ground. In other words, capillarity can be exploited to provide a level of reliable control in space in a similar way that gravity does on earth—even for impure, poorly wetting, effectively contaminated liquids.

#### Application

So how does such a plumbing outcome enable space exploration? It identifies a path forward for the passive control of poorly wetting liquids using container shape alone. Perfectly wetting liquids have long been addressed using a wide variety of liquid acquisition devices^16^, but the Space Cup approach is successful for all wetting liquids and does not rely on internal devices to create the local capillary pressure gradients needed to control the liquid. This applies to open, closed, flexible, and rigid containers alike. With these design tools in hand, scaled versions of such containers may be prototyped for low-g testing in short duration drop towers on earth, which was accomplished for 1 cm scale Space Cups in a 2.1 second tower^12,17^. This approach boosts TRL and reduces the time and costs of development and verification dramatically.

**Fig. 4:**
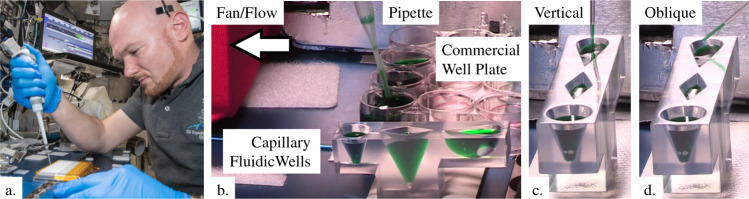
Pipetting with well plates on ISS. **a** German ESA astronaut A. Gerst pipetting in an open cabin of ISS in support of Protein Crystal Growth Experiments. **b** Gerst conducting STCE pipetting and stability tests on ISS using commercial well plates and low-g custom 3D printed capillary fluidic wells consisting of a tapered cone, rhombus, and “space cup” geometries (left to right with green-dyed water). Images of **c** vertical and **d** oblique tests. All well plates performed adequately with the capillary fluidic wells demonstrating greater fluid control and stability. Though not observed by eye, it is expected that nearly all pipette withdrawals resulted in spurious satellite droplet ejections, the majority of which are readily captured by local fan airflow identified in **b**.

Reusable cups aboard spacecraft could eliminate the need for thousands of drink bags over a long-duration mission. But the value of special container geometry passively controlling the fluid behavior in space as reliably as on earth bids well for numerous spacecraft applications. As an example, the need for fast and safe wet lab unit operations on ISS has increased dramatically with efforts to conduct high throughput biological experiments, sequence DNA samples, map the biome of ISS, and more. The work frequently requires numerous sample preparation and extraction steps, which in turn requires manual or automated pipetting operations (i.e., injecting, mixing, withdrawing liquid samples) from pre-sealed standardized well plates containing liquid reagents covered with un/coated metalized films or foils. A commercial 96-well microplate is shown in Fig. 4a and b during pipetting demonstrations on ISS as part of the Surface Tension Containment Experiment^18^ (STCE) to be described in greater detail in Section Rivulet rupture, pipetting, and slime in space.

The low-g configuration of the liquid reagents in the sealed well plates is critical. On earth, gravity simply orients the liquid over the base of the wells when oriented horizontally. However, in a low-g environment, the liquid can cover the lid such that it is flung away when the foil is removed or is ejected out upon pressurization during a pipette puncture and insertion. The uncontrolled release of such reagents could be hazardous in low-g where spurious droplets simply fly away without falling as will be discussed in Section Rivulet rupture, pipetting, and slime in space. Such ill-effects may be readily avoided with a “Space Cup”-type well-plate solution. Capillary considerations in the geometry of the well plate assure that liquid covers the base of the well. The liquid is not removed when the lid is, and the well is not unfavorably pressurized if the lid is punctured—the capillary geometry of the well plate in space provides the reliability of well plates on earth. Other geometries can be designed with similar outcomes in mind. Such stable liquid control was demonstrated aboard the ISS as part of the STCE demonstrations for conical, rhombic, and “Space Cup” wells, Fig. 4c. Numerous scaled model drop tower tests corroborate these findings.

### Rivulet rupture, pipetting, and slime in space

Liquid rivulets are ubiquitous in nature and industry. They form nearly every time a fluid body ruptures or is detached from a wetted wall or disconnected from a wetting liquid (i.e., itself). For example, in the case of pipetting, when a pipette is removed from a liquid sample following either an injection, stir, or withdrawal operation the wetting liquid is stretched between sample and pipette tip until the rivulet ruptures. On earth, within a fraction of a second, the rupture process produces microscale satellite and mother droplets that quickly fall back into the fluid body (or well plate as discussed in Section Coffee cups, fuel tanks, and well plates) due to gravity. The drops are usually too fast and too small to be observed with the unaided eye. Extensive terrestrial microfluidics research has been conducted on this subject due to its innate beauty and myriad industrial applications^19,20^.

However, when one pipettes aboard spacecraft, the potentially infectious or toxic satellite droplets ejected when a rivulet ruptures will simply fly away only to impact camera lenses, instrument panels, the crew, or be swept away into the cabin airflow until they either evaporate, impact something else, or are capture by the cabin air filters^21^. Many thousands if not tens of thousands of pipette operations have been conducted aboard the ISS. Hundreds of thousands more are likely. Since rivulets form in a vast number of additional fluids operations aboard spacecraft it is thus important to understand the nature of the phenomena in low-g such that simple robust capillary fluidic “plumbing solutions” can be established to mitigate them.

**Fig. 5:**
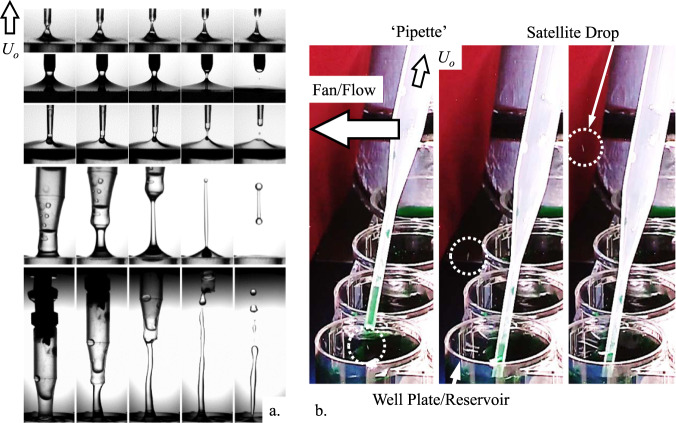
Filament rupture and droplet ejection during low-g pipetting from well plates. **a** Low-g drop tower demonstrations of distinct liquid ejections from rupturing rivulets following pipette withdrawal: (top to bottom) no “observed” ejection, “Satellite” droplet ejection, “Mother” droplet, “Inertial Ligament,” and large Inertial Ligament ejection. **b** ESA astronaut A. Gerst pipetting aboard the ISS with no visible evidence of droplet ejection but magnified slow-motion observations reveal satellite droplet ejection (barely visible streaks within the white dashed circles) during nearly every pipette tip withdrawal event.

It is well-known that “satellite” and “mother” droplets often form when rivulets rupture on earth^19^. Because static rivulets on earth are essentially limited in radius *R* and length *L* by gravity *g*, where *R*·*L*≲ *σ*/*ρg*, with *σ* the liquid surface tension and *ρ* the liquid density, one might wonder if such relationships hold as *g* approaches zero and rivulet sizes grow to enormous unearthly proportions. As shown in Fig. 5a, for a variety of pipette removal tests performed in a drop tower^22,23^, satellite droplets, mother droplets, and low-g unique inertial ligaments form routinely in low-g environments—somewhat similarly to on earth but over significantly larger length scales. [We note that, though not necessarily on purpose, satellite droplet ejection during large liquid bridge rupture events have been observed in space experiments^24–26^.] The ejected droplet size distributions, speeds, and trajectories depend on pipette dimensions, withdrawal rate, and fluid properties, but not on gravitational draining or ballistics common to terrestrial rivulets. We note that on earth, due to draining, such rivulets can rupture at the pipette tip often sending the ejected droplets statistically downward. In low-g, such rivulets typically rupture at the liquid reservoir often sending the ejected droplets statistically upward.

**Fig. 6:**
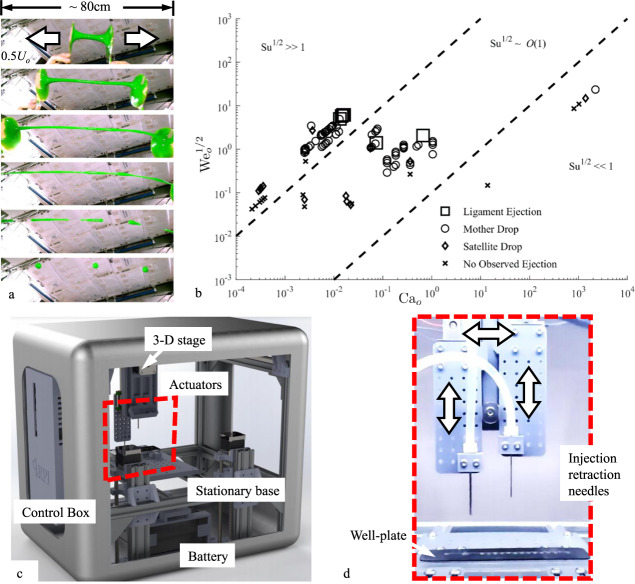
Low-g rivulet rupture and automated test apparatus. **a** Approximately 150 mL blob of Nickelodeon Slime was stretched into a rivulet between two ping pong paddles at velocity *U*_*o*_ ≈ 1 m/s by Italian ESA astronaut L. Parmitano. The length of the rivulet before rupture is >1 m and the five mother droplet diameters are ≈ 3 cm. **b** Drop tower and ISS data regime map of low-g “aqueous” rivulet rupture in terms of dimensionless inertia *We*_*o*_^1/2^ and viscous resistance *Ca*_*o*_. The Slime data resides in the low inertial domain *Su*^1/2^ << 1 with values of *Ca*_*o*_ at least three orders of magnitude higher than values attained on earth. **c** Automated pipetting, sample preparation, and sequencing experiment rig for high-rate rivulet rupture drop tower investigations and ejected droplet mitigation methods development with the magnified device shown in **d** red dashed region (courtesy IRPI LLC).

US astronaut Kate Rubins conducted thousands of pipetting operations in the open cabin of the ISS as part of the first DNA sequencing demonstration in space. Surprisingly, no satellite droplets were observed by both the Rubins and the cadre on the ground. Subsequently, as part of a specific flight demonstration effort^18,22,23^ to observe ‘expected’ satellite droplet ejections, German ESA astronaut Alexander Gerst conducted hundreds of pipette withdrawal operations at higher camera magnification, as shown in Fig. 5b. Again, no spurious ejected droplets were observed by both Gerst and the cadre on the ground. However, upon post-flight analysis of the downloaded images, it was just possible to distinguish satellite droplets produced at the limits of the camera resolution and frame rate for nearly all pipette withdrawals. An example is provided in Fig. 5b. As on earth, for the pipette types employed, the small size and high speed of the droplets could not be observed by the unaided eye. However, through a windfall opportunity the crew of the ISS Nickelodeon “Slime in Space” outreach activity^22,27,28^, US astronauts Christina Koch and Drew Morgan, and Italian ESA astronaut Luca Parmitano performed ad-lib rivulet break-up demonstrations with a fluid ~20,000 times more viscous than water (‘Slime’) establishing up to meter-long rivulets and five each ≈ 3 cm diameter mother droplets. Such droplets are large and slow and easily observed by the eye as shown in Fig. 6a. In the figure, Parmitano squeezes approximately 150 mL of Slime between two “ping pong paddles” on ISS. He then separates the paddles by hand at a rate of ~ 1 m/s (ref. to fingers in top frame of Fig. 6a). The large length scale overdamped rivulet rupture is representative of pipetting and other rupture phenomena performed at microscales on ISS as well as on earth. See McKinley et al.^29,30^ for highly controlled ligament stretching phenomena in terrestrial and low-g environments, respectively. See also, ref. ^31^ for production of a “mother droplet” following the “rupture” of an irregular thixotropic “pudding rivulet” on ISS.

Extensive drop tower tests were conducted showing nearly universal ejection of droplets in low-g pipetting operations, a sample of which is presented in the rivulet rupture regime map of Fig. 6b as a function of the pipette removal parameters and fluid properties; Weber number *We*_*o*_ = *ρU*_*o*_^2^*R*_*o*_/*σ*, Capillary number *Ca*_*o*_ = *μU*_*o*_/*σ*, and Suratman number *Su* = *ρσR*_*o*_/*μ*^2^, where *U*_*o*_ is the rivulet separation velocity (i.e., pipette withdrawal velocity), *μ* is the liquid dynamic viscosity, and *R*_*o*_ is the initial radius of the rivulet (i.e., pipette tip radius). The legend of the figure denotes large-volume ligament ejections, intermediate-volume mother droplet ejections, tiny satellite droplet ejections, and conditions where droplet ejection, if it occurs, is below observable limits. The Nickelodeon Slime data is included in the figure. These results confirm that for sufficiently high withdrawal rates *We*_*o*_^1/2^, droplets are ejected with nearly every rivulet rupture event and that the ejected droplet radii can be estimated from such a map depending on the definitions of the various regimes^22^ with knowledge of fluid properties *σ*, *μ*, and *ρ*, pipette radius *R*_*o*_, and pipette withdrawal velocity *U*_*o*_.

#### Application

So how does such a plumbing outcome enable space exploration? It identifies a path forward for the passive control of myriad often imperceptible droplet ejections that arise during myriad routine though potentially harmful fluids management functions. As on earth, such potentially problematic satellite droplets are the products of numerous routine “plumbing” operations in addition to typical wet lab operations for experiments in space such as pipetting: e.g., fluids handling equipment replacement and repair (i.e., disconnecting liquid tubing, QDs, etc.), opening lids, syringe functions, plant and animal water management equipment, food and beverage preparation, washing for hygiene, and when lips detach from food items, drink bags, and even cups as discussed in Section Coffee cups, fuel tanks, and well plates. [It is well-known that terrestrial sinks and toilets produce thousands of such droplets^32^ arising from rivulet as well as bubble rupture, each droplet assuming a ballistic trajectory. The droplet ejection surface of an effervescent carbonated drink, to be introduced in connection with Fig. 10e, provides a related case in point.]

Gravity typically takes care of satellite droplet mitigation on earth—an extremely simple plumbing solution. Aboard spacecraft, alternate methods must be adopted. The choice of the low-g method stems naturally from knowledge of the expected low-g ejected droplet characteristics, speed, and trajectory, as briefly presented herein, i.e., Fig. 6b. In an attempt to avoid the use of an additional level of containment, practical local droplet collection might simply employ an absorbent cloth nearby, but it may also require control of the system to maintain droplet formation below detectable limits (i.e., the slow disconnection of tubing containing hazardous liquids as in Fig. 5a top), employment of convective airflow as successfully demonstrated in the example of Fig. 5b, or, say, control of system geometry such as pipette and well-plate size, shape, fill level, and wetting properties, as successfully demonstrated by Turner^22,23^ (i.e., depth, taper, etc.) as suggested in Fig. 4c, d. Other methods are considerable.

**Fig. 7:**
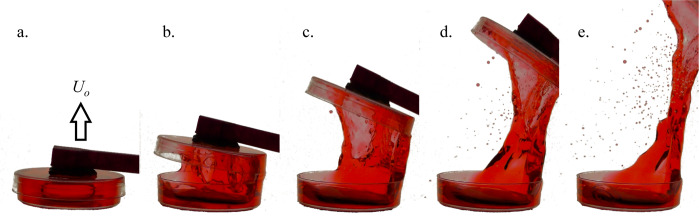
Simple removal of a Petri dish lid during a drop tower test. **a** Low-g equilibrium configuration, **b**, **c** lid removal produces films and rivulets that rupture producing hundreds of satellite droplets in **d**, **e**. The large rivulet at right in **e** again ruptures producing enormous spurious droplets^57^.

Passive mundane capillary fluidic plumbing solutions to such inadvertent droplet ejections reduce the need for inefficient additional levels of containment and improve process performance to levels that save significant crew time and spacecraft resources. Specifically, one might imagine the importance of such solutions for massively parallelized automated sample preparation and DNA sequencing operations aboard spacecraft. A terrestrial drop tower test version of such a robotic device capable of numerous operations per test is shown in Fig. 6c, d. However, in general, it is not difficult to identify simple processes that function flawlessly on earth, but disastrously in space—an example of which is shown in Fig. 7, where a standard Petri dish lid is removed in low-g producing small and ultimately large rivulets that rupture into hundreds of satellite droplets varying in volume by over three orders of magnitude.

### Low-g flows in sealed chambers, open channels, and in-line bubble phase separators

**Fig. 8:**
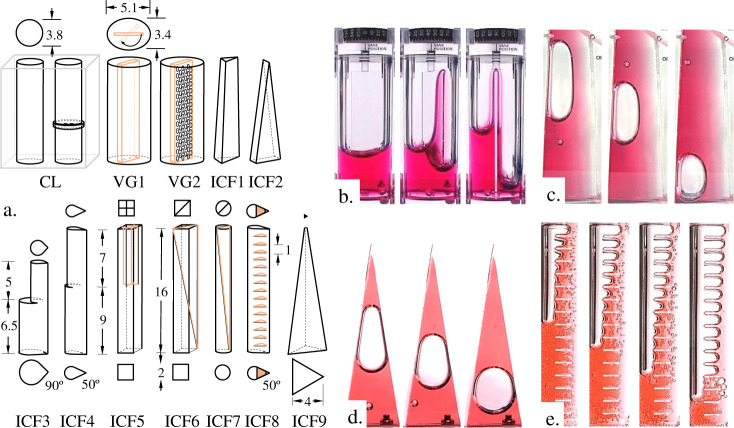
Selection of Capillary Flow Experiment (CFE) test sections and ISS results. **a** Sketches of fluid bearing chambers for ISS capillary fluidics experiments CFE and CFE-2 to approximate scale with dimensions in cm: moving Contact Line dynamics and capillary stability (CL), critical geometric wetting in an elliptical cylindrical container with pivoting Vane with Gap between wall and vane (VG), and ullage migration due to Interior Corner Flow (ICF). **b** Flight images of static interface configurations in VG1 demonstrating critical geometric wetting and dynamic images in **c** ICF1, **d** ICF2, and **e** ICF8 demonstrating ullage migration with passive bubble phase separations.

Despite an extensive ISS assembly schedule, the Space Shuttle Columbia accident, limited American crew transport capabilities, shifting funding objectives, and so on, NASA never ceased to support a broad range of fundamental and applied microgravity research including fluid physics. From crew-tended handheld experiments to automated ground-controlled experiments, a wealth of capillary fluidics data has been and is being collected that now serves as a resource from which to draw capillary solutions to address persisting plumbing challenges aboard spacecraft^8,33^. Again choosing from examples with which the authors are most familiar, the handheld Capillary Flow Experiments^34,35^ (CFE, CFE-2) pursued contact line dynamics, capillary stability, critical geometric wetting, passive capillary migration in geometric families of containers, passive bubble phase separations, draining, and more. Sketches of the 13 vessels are shown in Fig. 8a with select images during operations provided in Fig. 8b–e. The experiments employed perfectly wetting PDMS fluids which in many cases could be accurately modeled via theoretical and numerical analyses^36–41^. These data are publicly available for download at the NASA archive for CFE (http://psi.nasa.gov/).

**Fig. 9:**
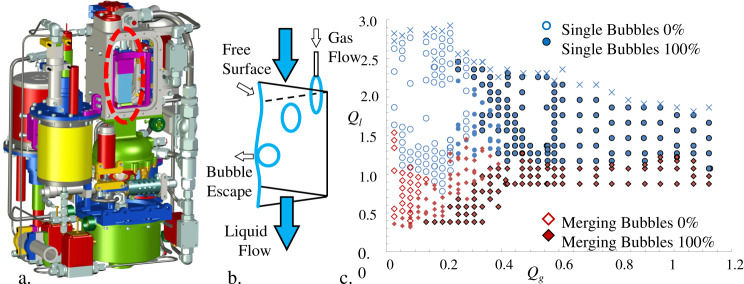
The Capillary Channel Flow (CCF-EU2) experiment with sample data. **a** Solid model of CCF with an open wedge test cell circled in red. **b** Simplified schematic of open wedge channel depicting passive low-g capillary separation mechanism of a single bubble. **c** Sample bubble phase regime map as function of liquid *Q*_*l*_ and gas flow rate *Q*_*g*_ with single and merged bubble regimes identified with either 0% or ~100% passive bubble separations.

Related experiments that focused on forced capillary flow phenomena were pursued as part of the automated ground-commanded Capillary Channel Flow experiment^42–44^ (CCF). One of the objectives of CCF was to study the nature of passive bubble migration in forced capillary flows of a perfectly wetting liquid through an open wedge conduit^45^. A partial CAD model of part of the CCF experiment hardware is shown in Fig. 9a, with a schematic of the simple test channel provided in 9b, and a sample bubble-phase regime map provided in 9c. The sketch of Fig. 9b shows how gas injection near the channel vertex pinches off single bubbles that are partially confined in the interior corner and driven by capillary forces in a crossflow direction. If the bubbles are large enough and the liquid flow slow enough, the bubbles reach the free surface, coalesce with it, and leave the flow. As seen from 9c, the slower the liquid flow, the more likely that single bubbles collide, merge, and leave the flow. Tens of thousands of data points were collected over several months of around-the-clock testing on ISS identifying flow conditions that identify regimes where the channel separates “~100%” of the incoming gas in the form of bubbles that escape the channel through the liquid free surface. These data are publicly available for download at the NASA archive for CCF (http://psi.nasa.gov/). It is immediately obvious under what flow conditions such channels can perform bubble phase separations in a manner much like bubbles rising in channel flows on earth, only aboard spacecraft do the surface tension, wetting, and channel geometry conspire to replace the role of gravity in the process.

**Fig. 10:**
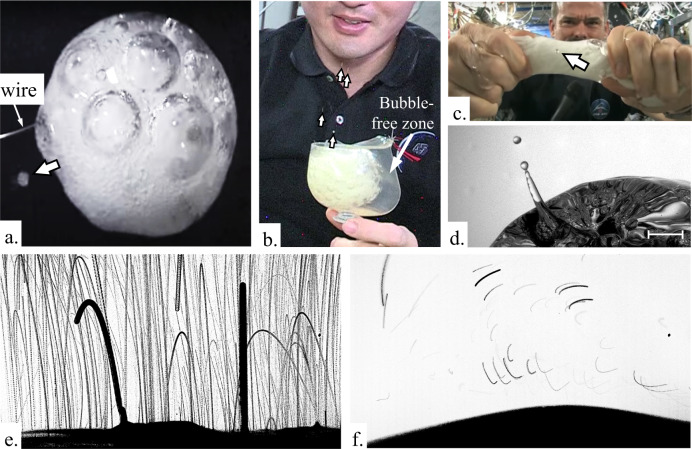
Droplets ejected from low-g bubble bursts (identified by arrows or tracks). **a** Bursting CO_2_ bubbles from a dissolving antacid tablet eject droplets from a 65 mL water blob suspended from a wire hoop by US astronaut D. Pettit on ISS^49^. **b** Similarly ejected droplets from similarly bursting CO_2_ bubbles in the Space Cup which confines the bubbles away from the primary capillary flow interior corner region of the cup as demonstrated by K. Lindgren. **c** Ejected droplet(s) from bursting bubbles as Canadian astronaut C. Hadfield wrings out a partially water-saturated cloth^51^. The ejected droplet identified impacts the camera lens. **d** Single image of a ‘large’ ejection following a bubble burst within an inertial vertical up flow of effervescing sparkling water during a low-g drop tower test^74^. An ~ 2 cm wide FOV of the similar test as shown in **d** is shown in **e**, **f**, where image overlays at 2125 fps are presented **e** before and **f** after the step reduction in buoyancy during a drop tower test.

Bubble separations of “~100%” are reported^45,46^ due to the high likelihood of imperceptible droplets ejected during bubble coalescence or burst phenomena—droplets that are below the detection limits of the imaging equipment. As discussed in connection with rupturing rivulets in Section Rivulet rupture, pipetting, and slime in space, such droplets are normally not visible by the unaided eye. However, a large terrestrial literature exists on this often microscale phenomenon made possible by high-speed video microscopy^47,48^. In low-g environments where bubbles can be enormous, large coalescing, merging, or otherwise bursting bubbles eject large liquid volumes in the form of flung sheets and films, geysers, and droplets. Figure 10a–c provides observations of such ejections which have been observed by astronaut crews for decades^49–51^ but have not received experimental attention to date. A single low-g “large” bubble rupture event is shown in Fig. 10d during a drop tower test employing effervescent flavored sparkling water. A wider camera angle of the phenomena is shown in Fig. 10e, f which presents overlain sequences of images before and after the step reduction in gravity during the drop tower test. The rate of bubble coalescence is greatly reduced in low-g due to the reduction in buoyancy that drives the bubbles ever upward toward the surface, Fig. 10e. Such buoyancy-driven phenomena occur in microgravity environments, but may be masked by other more efficient means of rupture such as bubble confinement and flow inertia (other mechanisms are noteworthy, i.e., thermo-, soluto-, acousto-, electro-capillary, etc^8^).

#### Application

So how do such microgravity fluids research outcomes inspire plumbing solutions that in turn enable space exploration? They identify design guides that may be applied to the innovation of plumbing elements for the passive control of perfectly wetting liquids using container shape only—in a manner akin to terrestrial solutions. In connection with the limited examples of this paper, the passive capillary fluidic bubble phase separation nature of the wedge or interior corner flows of CFE and CCF has led to spacecraft technologies for passive collection and separation of non-ideally wetting urine^46,52^, aerated hydroponic plant watering systems^53^, a de-bubbler for bio-fluid samples^54^, degassing “thin-film” liquid sorbent CO_2_ technologies^55,56^, and in-line bubble traps and phase separating devices^54^. Even the Space Cup highlighted in Section Coffee Cups, Fuel Tanks, and Well Plates employs the interior corner geometry to exude bubbles during drinking in a passive manner, as shown in Fig. 10b.

When practicable, such passive capillarity-driven separations are ideal because they have no moving parts and may be fabricated easily despite complex shapes by 3D printing. Such devices may be added to existing systems as in-line elements that do not add to system pressure losses. Applied in these ways, the reliability of flight systems that adopt such solutions are likely to gain significant reliability—even if such passive methods are added to existing mechanically pumped methods which could still function, though at a perhaps reduced level, should the primary mechanical system function fail. Perfect wettability is a requirement for some, but not all, designs as the above-cited applications suggest.

### Water ping pong, wastewater transport, and processing

**Fig. 11:**
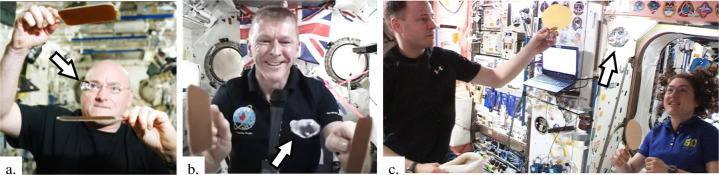
Demonstrations of super-hydrophobic surfaces by ISS crew. ISS astronauts **a** S. Kelly^58^, **b** T. Peake^59^, and **c** N. Hague and C. Koch^28^ demonstrate large length scale non-wetting phenomena with super-hydrophobic paddles and water blobs of ~ 10, 30, and 250 mL, respectively (see white arrows).

**Fig. 12:**
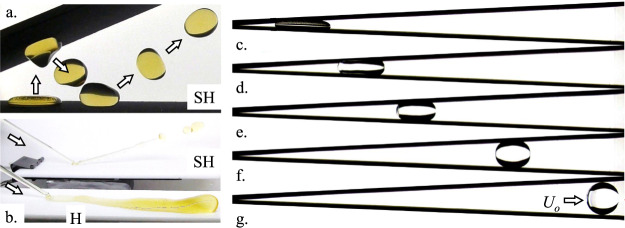
Drop tower test demonstrations of large length scale super-hydrophobic (SH) capillary phenomena. **a** Time overlay of 1 mL urine ersatz puddle jump^60^ rebounding between non-parallel plates. **b** Simultaneous drop tower tests of two 6 mL/s oblique jet impacts of ersatz urine on SH and H (hydrophobic, non-wetting) substrates illustrate the ease with which SH substrates repel the jet (courtesy IRPI LLC). The simply hydrophobic (H) surface does not yield rebound, but a wall-bound rivulet. We note also that such rebound phenomena do not occur at 1-*g*_*o*_ for the flow rates and properties of typical human urination. **c**–**g** The spontaneous ejection of a 5 mL droplet from a water puddle^65^ between SH tilted plates at 5°: **c** static 1-*g*_*o*_ interface, **b**–**f** capillarity-driven acceleration, and **g** non-oscillatory ejection at steady velocity *U*_*o*_ ≈ 10 cm/s.

US astronaut Scott Kelly^57,58^, UK ESA astronaut Tim Peake^59^, and most recently US astronauts Christina Koch and Nick Hague^28,60^ are shown in Fig. 11 manipulating various blobs of aqueous solutions with “ping pong” paddles on ISS. The surfaces of the paddles are highly non-wetting. Specifically, exploiting the combined effects of architectured surface roughness (laser etching) on a non-wetting substrate (i.e., PTFE), the paddles are “super-hydrophobic” in that wetting contact angles are ≳150° for water with little contact angle hysteresis^61^. Playing ping pong with water in space may be fun, but it also demonstrates several desirable practical behaviors: (1) the wide majority of dynamic droplet impacts result in 100% rebounds as an air layer envelops the water during the process preventing actual contact^62^, (2) the phenomena is enhanced in a low-g environment where “g-jitter” is ample enough to maintain perpetual blob motion with very little time spent in contact with the walls of the container, (3) droplets, slugs, rivulets, and jets^63^ simply and cleanly rebound from such super-hydrophobic surfaces inspiring non-contact fluids handling methods, and (4) free liquid bodies may be easily positioned without physically touching them—establishing a measure of containerless processing. Several further demonstrations from low-g drop tower tests are present in Fig. 12 for droplet rebound between plates^60,64^, jet rebound from a monolithic super-hydrophobic PTFE substrate yet no rebound from simple non-wetting substrate^60^, and large droplet ejection between tilted super-hydrophobic plates^65^.

The liquid is in effect passively repelled from these substrates which may be exploited to create a “top” or an “up,” in turn enabling more earth-like conceptions. It does not take much imagination to appreciate how such surfaces could be exploited as part of numerous plumbing solutions aboard spacecraft. The challenge is to create robust super-hydrophobic surfaces that are difficult to contaminate, regenerable if contaminated, resistant to wear, applicable to a wide range of geometries, and spaceflight certifiable. Specific applications will demand specific requirements, some of which may be met with current capabilities.

#### Application

So how does such a plumbing outcome enable space exploration? It identifies a passive means of capillary fluidic control: super-hydrophobic surfaces repel many aqueous solutions. Thus, such containers and conduits containing such fluids can do so in an essentially non-contact manner. For example, consider the plumbing elements of a spacecraft urine collection system. From the receptacle to the transfer hose to the separation and storage subsystem, super-hydrophobic plumbing materials could extend the life of such systems by dramatically reducing fluid-container contact, in turn reducing system contamination, fouling, odors, caustic pre- or post-treatment chemicals, flush procedures and resources, number of replacement parts, and crew hours consumed for maintenance. Further improvements in reliability are achieved due to the inherent simplicity, absence of moving parts, and the enhanced non-wetting nature of such surfaces in the low-g environment for small or large liquid inventories.

To our knowledge, super-hydrophobic surfaces are yet to find application in low-g plumbing solutions aboard spacecraft beyond the ping pong paddles. As part of a “no-touch” urine processor, Rasheed^66^ and Rasheed et al.^67^ report low-g drop tower demonstrations of super-heated super-hydrophobic Leidenfrost conduit capable of minimal contact for on-demand urine distillation. The nearly solid-state solution is compact, without moving parts, resists fouling, and does not require pretreatment chemicals. It does however require robust super-hydrophobic substrates that are compatible with the water reprocessing cycle as well as the entire spacecraft life support system. For example, silicate-based super-hydrophobic coatings may dissolve into the water system or become airborne, fouling other downstream plumbing elements as has been reported for other silicate sources leading to the fouling of an EMU valve^68^ and HVAC condenser surface (HVAC condensers spitting surface fouling). Non-silicate monolithic super-hydrophobic materials offer a safer choice. Such surfaces are under development for spacecraft applications, with a first prototype substrate sent to the ISS for testing in 2021. The demonstrations are part of the Collapsible Contingency Urinal technology development^69,70^ and will expose monolithic super-hydrophobic PTFE surfaces urine ersatz and actual urine streams. Further flight demonstrations relating to gas-water separations, water management for plant habitats, and wastewater processing are under pursuit.

## Summary

The design of standard plumbing elements for the fluids systems aboard spacecraft would be trivial if not for the nearly weightless environment. Unfortunately, even the simplest of plumbing components such as fittings, valves, conduits, and containers can perform poorly if the unique unearthly behavior of bubbles, films, rivulets, droplets, and large fluid interfaces in the microgravity environment are not understood. Such behaviors must be first anticipated to be either exploited or avoided, i.e., the Space Cup (Section Coffee Cups, Fuel Tanks, and Well Plates) exploits capillary phenomena to perform its task passively, while special considerations might be required to avoid interfacial instability, break-up, and, say, droplet ejections due to bubble or rivulet rupture (Sections Rivulet Rupture, Pipetting, and Slime in Space and Low-g Flows in Sealed Chambers, Open Channels, and In-Line Bubble Phase Separators).

In this paper, we highlight several examples of spaceflight capillary fluidics research and engineering demonstrations that shed light on passive gravity-free fluid control, rivulet rupture, bubble separations, and the potential of highly non-wetting surfaces. These phenomena have broad cross-cutting application to many of the remaining fluid challenges for plumbing systems aboard spacecraft. Experiential knowledge of each would serve the young microgravity fluid systems designer well. Such knowledge no longer needs to be rare. A deeper understanding of the potentially mission-enabling phenomena is achieved by exposure to experiments and demonstrations conducted to date, a grounding in the terrestrial literature, an understanding of the degree to which scaling laws extend to the low- and variable-g environments, and an ability to design and thoroughly test in such environments (i.e., drop tower, spacecraft, etc.). Examples of our shortcomings abound (HVAC condensers spitting surface fouling)^71–73^. The reliability of mundane plumbing systems is central to life support aboard spacecraft, and reliability on par with terrestrial plumbing systems enables the safer human exploration of space.

We conclude with a summary of the basic capillary fluidics sub-topics highly valued for the design of simple, yet advanced, plumbing systems for spacecraft^8^: interfacial configurations and stability, metastable states, time constants, natural frequencies, free-, pinned-, and mixed-boundary conditions at the contact line, bubble and droplet dynamics, mergers and coalescence, the combined effects of wetting and geometry, the limits of multiphase flow regimes in plumbing elements, scaling laws, and numerical tools for efficient static and dynamic modeling. These topics are foundational to the low-g demonstration and application of the four theme problems highlighted herein.
